# Role of the Host Membrane Trafficking Protein Dynamin 2 in Cell-to-Cell Spread of Bacterial Pathogens

**DOI:** 10.3390/cells15110994

**Published:** 2026-05-28

**Authors:** Keith Ireton

**Affiliations:** Department of Microbiology and Immunology, University of Otago, Dunedin 9054, New Zealand; keith.ireton@otago.ac.nz

**Keywords:** cell-to-cell spread, *Listeria monocytogenes*, *Shigella flexneri*, *Burkholderia thailandensis*, dynamin 2 GTPase

## Abstract

**Highlights:**

**What are the main findings?**

- The host membrane trafficking protein Dynamin 2 is manipulated by the bacteria *Listeria monocytogenes*, *Shigella flexneri*, and *Burkholderia thailandensis* to promote the intercellular spread of these pathogens in human tissues.
- Control of Dynamin 2 by these three bacteria occurs through different mechanisms, which affect distinct steps in the intercellular spreading process.

**What are the implications of the main findings?**

- Although evolutionarily distant, the bacteria *L. monocytogenes*, *S. flexneri*, and *B. thailandensis* have each developed mechanisms to target Dynamin 2 to enhance their cell-to-cell spread.
- Strategies that interfere with these pathogens’ abilities to target Dynamin 2 may prove useful in development of therapeutics.

**Abstract:**

Although evolutionarily distant, the bacteria *Listeria monocytogenes*, *Shigella flexneri*, and *Burkholderia thailandensis* each undergo a “cell-to-cell” spreading process that allows these pathogens to disseminate within human tissues. Spread initiates when bacteria polymerize actin filaments that propel them through the host cell cytosol. The pathogens then remodel the plasma membrane into protrusions that are internalized by adjacent cells and resolved into double membranous vacuoles (DMVs) which lyse to liberate bacteria. In this review, we discuss recent publications indicating that *L. monocytogenes*, *S. flexneri*, and *B. thailandensis* each enhance their spread by altering the subcellular localization of human Dynamin 2—a GTPase that regulates endocytosis and other trafficking pathways. Interestingly, Dynamin 2 controls distinct steps in spread of *L. monocytogenes*, *S. flexneri*, and *B. thailandensis*. In the case of *L. monocytogenes*, the GTPase has the potential to restrict protrusion formation by generating tension at tight junctions. However, *L. monocytogenes* secretes a protein that relieves this restriction of protrusions, allowing efficient spread. During dissemination of *S. flexneri* and *B. thailandensis*, Dynamin 2 is co-opted to resolve protrusions into DMVs. *B. thailandensis* also mobilizes Dynamin 2 to lyse DMVs. These findings highlight diverse ways in which bacteria control Dynamin 2 to augment spread.

## 1. Introduction

Several intracellular bacterial pathogens, including *Listeria monocytogenes*, *Shigella flexneri*, members of the spotted fever group (SFG) of *Rickettsia*, and *Burkholderia* spp. undergo a “cell-to-cell spread” process that allows bacteria to disseminate from infected human cells to neighboring cells without leaving the host cytosol [1,2,3]. Intercellular spread is thought to benefit bacteria by providing protection from the humoral immune response and extending access to nutrients by joining together the cytoplasm of infected host cells [4,5,6].

**Figure 1:**
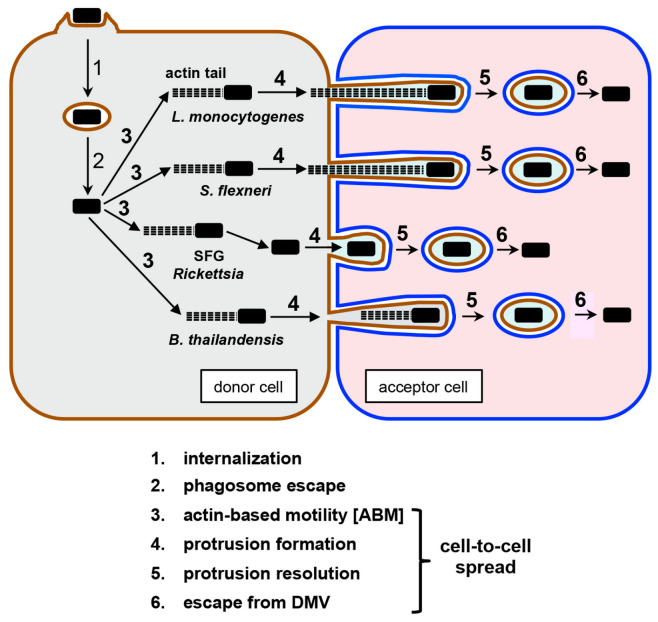
Intracellular life cycles of *L. monocytogenes*, *S. flexneri*, SFG *Rickettsia*, and *B. thailandensis*. After internalization into human cells and escape from phagosomes, these bacteria spread between cells through actin-based motility, generation of plasma membrane protrusions that protrude into neighboring cells, resolution of protrusions into DMVs, and disruption of DMVs to access the cytosol of protrusion-accepting cells. Adapted from Dowd et al. 2021 [1] with permission from Elsevier, 2026.

*L. monocytogenes*, *S. flexneri*, SFG *Rickettsia*, and *Burkholderia* spp. all have the ability to induce their internalization into human cells and escape membranous phagosomes to access the cytosol of the host cell (Figure 1, steps 1 and 2) [7,8,9,10]. Spread is initiated when cytosolic bacteria polymerize host actin monomers into filaments, resulting in tail-like structures that propel pathogens through the cytoplasm of human cells (Figure 1, step 3). This process is termed “actin-based motility” (ABM) [2]. After reaching the cell periphery, motile bacteria remodel the host plasma membrane into protrusions that project into neighboring cells (Figure 1, step 4). In this review, we refer to host cells that produce protrusions as “donors” and cells that engulf these structures as “acceptors”. The importance of plasma membrane protrusions in mediating cell-to-cell spread of *L. monocytogenes*, *S. flexneri*, and SFG *Rickettsia* has been long appreciated [11,12,13,14,15]. These structures are resolved into double membranous vacuoles (DMVs) from which bacteria escape into the cytoplasm of the acceptor cell (Figure 1, steps 5 and 6). Early studies with *Burkholderia* spp. indicated that these bacteria induce the formation of multinucleated giant cells (MNGCs) [16,17,18]. Intercellular spread was therefore thought to occur through cell–cell fusion. However, recent work reveals that, like *L. monocytogenes*, *S. flexneri*, and SFG *Rickettsia*, the *Burkholderia* species *B. thailandensis* forms plasma membrane protrusions that contribute to spread [19,20].

**Figure 2:**
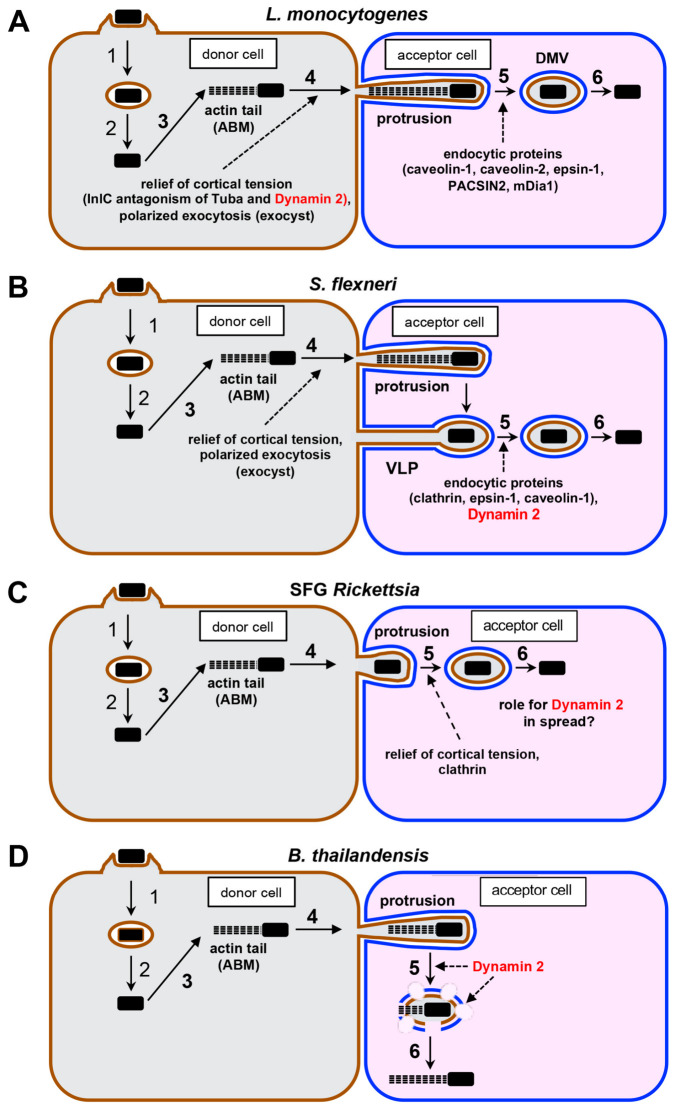
Roles of host membrane trafficking proteins in cell-to-cell spread of *L. monocytogenes*, *S. flexneri*, SFG *Rickettsia*, and *B. thailandensis*. Steps 3, 4, 5, and 6 indicate the protrusion formation, protrusion resolution, and DMV formation stages of spread, respectively. (**A**) To increase the efficiency of protrusion formation, *L. monocytogenes* produces the protein InlC, which antagonizes human Tuba and Dynamin 2 proteins to relieve tension at the cell cortex [24,38]. *Listeria* also manipulates the host exocyst complex to provide membrane for protrusion generation [26]. The human endocytic proteins caveolin-1, caveolin-2, epsin-1, PACSIN2, and mDia1 contribute to the resolution of protrusions to DMVs [28,29,31]. (**B**) *S. flexneri* shares with *L. monocytogenes* the ability to dissipate cortical tension and exploit the host exocyst to promote protrusion formation [25,27]. Protrusions are converted to VLPs, which are then resolved to DMVs through the endocytic proteins clathrin, epsin-1, caveolin-1, and Dynamin 2 [30,40]. (**C**). In the case of SFG *Rickettsia*, actin tails that mediate ABM are lost when bacteria reach the periphery of the human cell to make protrusions [12]. Resolution of protrusions to DMVs involves bacterial-induced relief of cortical tension in donor host cells and activity of the endocytic protein clathrin in acceptor cells [12,32]. (**D**). *B. thailandensis* produces protrusions that are converted into structures resembling DMVs in a manner dependent on host Dynamin 2. This GTPase also promotes the lysis of the DMV-like structures.

The mechanisms of ABM of *L. monocytogenes*, *S. flexneria*, SFG *Rickettsia*, and *Burkholderia* spp. have been studied for 10–30 years and are understood at a high level of detail [2]. More recent results have shed light on how protrusions form and resolve into DMVs from which bacteria escape [1,3,21,22,23]. These findings indicate that *L. monocytogenes*, *S. flexneri*, and SFG *Rickettsia* each relieve cortical tension at the cell periphery, which results in increased frequency of formation of protrusions or conversion of these structures to DMVs (Figure 2A–C) [12,24,25]. In addition, several host proteins with established roles in exocytic or endocytic membrane trafficking were shown to control protrusion formation or resolution. For example, *L. monocytogenes* and *S. flexneri* both stimulate polarized exocytosis through the host exocyst complex to enhance the generation and elongation of protrusions (Figure 2A,B) [26,27]. In addition, the human endocytic proteins clathrin, caveolin-1, caveolin-2, epsin-1, mDia1, and/or PACSIN2 contribute to the resolution of protrusions made by *L. monocytogenes*, *S. flexneri*, and/or SFG *Rickettsia* (Figure 2A–C) [28,29,30,31,32].

The roles of cortical tension, the exocyst complex, and endocytic proteins in cell-to-cell spread of *L. monocytogenes*, *S. flexneri*, and SFG *Rickettsia* have been discussed in several recent reviews [3,21,22,23,33]. In this article, we focus specifically on publications in 2024 and 2025 that examine the function of the human GTPase Dynamin 2 in the intercellular spread of *L. monocytogenes*, *S. flexneri*, and the *Burkholderia* species *B. thailandensis*. Dynamin 2 is a ubiquitously expressed protein that promotes clathrin- and caveolin-dependent endocytosis, exocytosis, autophagy, and actin polymerization [34,35,36,37,38]. Recent studies show that Dynamin 2 contributes to cortical tension that potentially restricts protrusion formation of *L. monocytogenes*, but this bacterium overcomes this restriction through the action of a secreted virulence protein called InlC (Figure 2A) [39]. In the case of *S. flexneri*, Dynamin 2 is recruited to specialized structures called “vacuole-like protrusions” (VLPs), which the GTPase helps resolve into DMVs (Figure 2B) [40]. During infection by *B. thailandensis*, the most frequently observed spreading mechanism involves detachment of protrusions from the plasma membrane, followed by lysis of the detached structures, which resemble DMVs (Figure 2D) [20]. Both protrusion detachment and lysis are controlled by Dynamin 2. Whether Dynamin 2 GTPase affects spread of SFG *Rickettsia* has yet to be reported (Figure 2C).

The recent publications described above led to the surprising revelation that Dynamin 2 can regulate distinct steps in cell-to-cell spread of different bacterial pathogens. However, mechanisms through which *L. monocytogenes*, *S. flexneri*, and/or *B. thailandensis* disrupt or manipulate Dynamin 2 function to promote their spread are not well understood. We begin this review by describing the known biological functions of Dynamin 2 in mammalian cells. We then discuss publications by Tijoriwalla et al. (2024) [39], Rolland et al., (2025) [40], and Plum et al. (2024) [20], highlighting their key findings and questions to be answered in future work.

## 2. Biological Functions and Structure of Dynamin 2

The mammalian Dynamin family of GTPases comprises three proteins. Dynamin 1 and Dynamin 3 are expressed mainly in the brain, whereas Dynamin 2 is ubiquitously expressed [34,41]. While all three of these Dynamin proteins promote membrane fission during clathrin-mediated endocytosis, Dynamin 2 is also needed for caveolae-dependent endocytosis and other membrane trafficking processes, including exocytosis and scission of autophagosome precursors from the recycling endosome (RE) [34,35,36,37,42]. In addition, Dynamin 2 regulates the microtubule cytoskeleton to control dynamic instability and the actin cytoskeleton to regulate cell migration, myoblast formation, tight junction structure, and production of protrusive structures such as podosomes, dendritic spines, and axonal growth cones [35,38,42,43,44,45,46,47,48,49].

**Figure 3:**
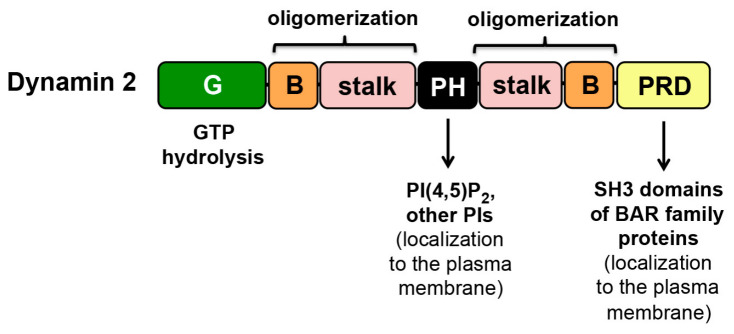
Structure of Dynamin 2. Domains involved in GTP hydrolysis, oligomerization, binding to phosphoinositides (PIs) or interaction with SH3 domains are depicted. “B”, “PH”, and “PRD” indicate the bundle signaling element, pleckstrin homology domain, and proline-rich domain, respectively.

Like all Dynamin family proteins, Dynamin 2 consists of an amino-terminal GTPase (G) domain, a bundle signaling element (BSE), a central stalk region comprising a four-helix bundle, a pleckstrin homology (PH) domain, and a carboxyl-terminal PRD region with proline-rich sequences (Figure 3) [42]. GTP hydrolysis by the GTPase domain induces conformational changes in the BSE that stimulate oligomerization of Dynamin 2 molecules on membranes due to interactions between the protein’s stalk domain. When oligomerized, the GTPase activity of Dynamin 2 increases by about 40-fold [50], allowing the protein to induce membrane fission during endocytosis and other trafficking processes. The PH domain in Dynamin 2 binds the lipid phosphatidylinositol 4,5-bis phosphate [PI(4,5)P_2_], which helps recruit the GTPase to the plasma membrane [42]. Membrane recruitment is also promoted by interaction of the PRD in Dynamin 2 with Src homology three (SH3) domains in proteins that have amino-terminal BAR (Bin/Amphiphysin/Rvs) domains, including amphiphysin, endophilin, syntaxin 9 (SNX9), and Tuba [39,51,52].

## 3. Role of Dynamin 2 in Protrusion Formation by *Listeria monocytogenes*

### 3.1. Dynamin-2-Dependent Restriction of Protrusion Formation Through Cortical Tension

**Figure 4:**
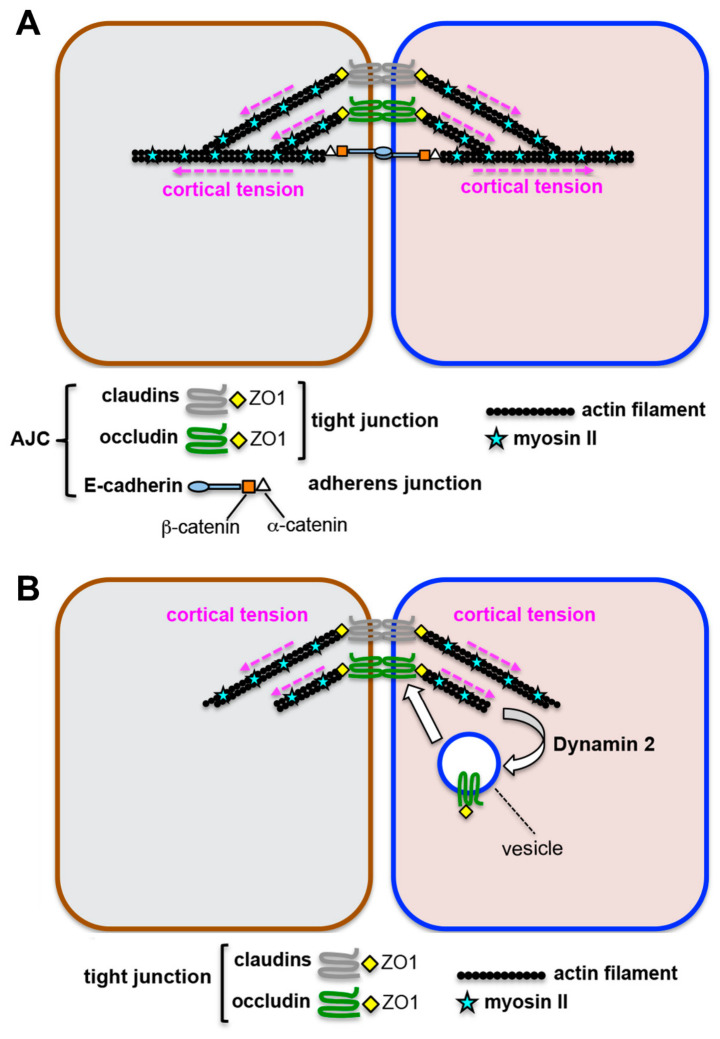
Cortical tension at the apical junctional complex (AJC). (**A**) Role of AJC components, actin filaments, and myosin II proteins in generating tension. At tight junctions, the transmembrane proteins claudins and occludin engage in homophilic interactions that seal cells together to form a paracellular barrier [54,57]. In adherens junctions, homophilic binding between E-cadherin molecules contributes to mechanical strength and maintenance of cell polarity [54,56]. Cytoplasmic domains in claudins or occludin interact with the scaffolding protein ZO-1, which anchors these transmembrane proteins to the actin cytoskeleton. Similarly, the cytoplasmic domain of E-cadherin is coupled to the actin cytoskeleton through a complex composed of β-catenin and α-catenin. Myosin II proteins interact with actin filaments at the AJC to generate cortical tension. (**B**) Dynamin 2 might control cortical tension by promoting endocytosis and recycling of tight junction proteins such as occludin. Such recycling is thought to be a homeostatic mechanism to maintain barrier function [58].

*L. monocytogenes* is a Gram-positive food-borne pathogen that causes gastroenteritis, central nervous system infections, and abortion [53]. This bacterium initiates disease by infection of cells of the intestinal epithelium. These cells form a tight barrier due the apical junctional complex (AJC), which comprises tight junctions and underlying adherens junctions (Figure 4A) [54]. The AJC is linked to an extensive network of actin filaments and myosin II proteins that generate contractile tension at the cell cortex, thereby controlling the barrier properties of the epithelium [55,56,57]. This tension poses a potential obstacle to cell-to-cell spread of *L. monocytogenes* and other pathogens that undergo ABM, as it could restrict their ability to remodel the host cell plasma membrane into protrusions. However, experiments with the polarized human epithelial cell line Caco-2 show that *L. monocytogenes* uses a secreted virulence protein called InlC to disrupt cortical tension, resulting in efficient intercellular spread [24].

**Figure 5:**
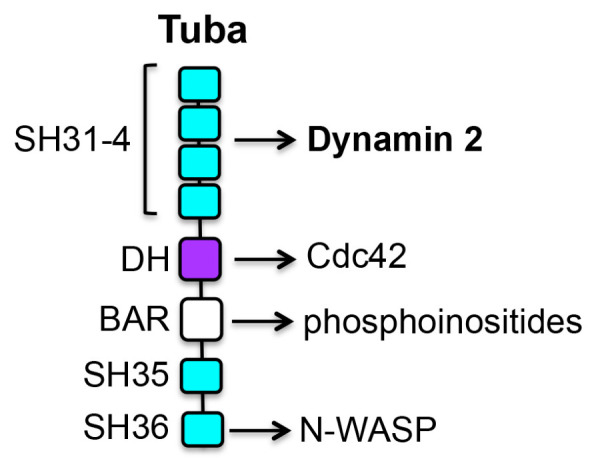
Structure of the human scaffolding protein Tuba. This cytosolic protein has 6 Src homology 3 (SH3) domains, a Dbl homology (DH) domain, and a Bin/amphiphysin/Rvs (BAR) domain [59]. Human binding partners of these domains are indicated to the right. Of relevance to this review article is the interaction of four tandem SH3 domains (SH31-4) in the amino terminus of Tuba with Dynamin 2 and binding of the carboxy terminal SH3 domain (SH36) to the actin regulatory protein N-WASP.

**Figure 6:**
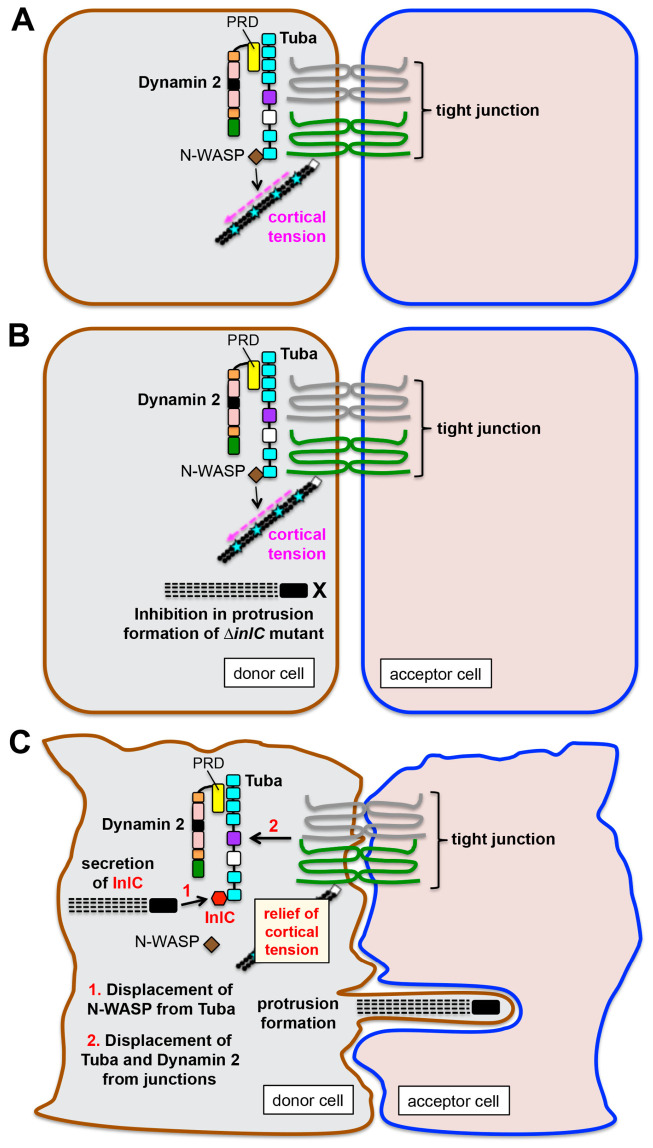
Control of cortical tension and intercellular spread of *L. monocytogenes* by Tuba and Dynamin 2. (**A**) In uninfected polarized Caco-2 cells, interaction of the SH31-4 region in Tuba with the PRD in Dynamin 2 recruits the GTPase to tight junctions. The SH36 domain in Tuba binds the actin nucleation-promoting factor N-WASP. Together, Tuba, Dynamin 2, and N-WASP promote cortical tension at right junctions. (**B**) Cortical tension restrains protrusion formation in Caco-2 cells infected with a mutant strain of *L. monocytogenes* deleted for the *inlC* gene. (**C**) In cells infected with wild-type *L. monocytogenes*, InlC protein is secreted and binds to the SH36 domain in Tuba. This interaction results in displacement of N-WASP from Tuba and also the dissociation of Tuba and Dynamin 2 from tight junctions. These events result in decreased cortical tension and enhanced protrusion formation by *L. monocytogenes*.

InlC relieves tension and enhances *L. monocytogenes* protrusion formation by antagonizing the human scaffolding protein Tuba [24,59]. Tuba localizes to tight junctions in the AJC and contains several functional domains, including a central BAR (Bin/Amphiphysin/Rvs) domain that binds plasma membrane phosphoinositides, a Dbl Homology (DH) domain that activates the GTPase Cdc42, and six Src Homology 3 (SH3) domains [60,61] (Figure 5). An SH3 domain at the carboxyl terminus of Tuba, referred to as “SH36”, interacts with the actin nucleation-promoting factor N-WASP [60]. This interaction promotes cortical tension at the AJC in uninfected polarized Caco-2 cells (Figure 6A) or in cells infected with a *L. monocytogenes* mutant strain deleted for the *inlC* virulence gene [24,61] (Figure 6B). A combination of biochemical, structural, and cell biological studies demonstrated that wild-type *L. monocytogenes* spreads more efficiently than an isogenic ∆*inlC* mutant strain because its InlC protein binds directly to the SH36 domain in human Tuba, thereby displacing N-WASP and reducing cortical tension (Figure 6C) [24,59].

Results published in 2024 show that the host GTPase Dynamin 2 acts together with Tuba and N-WASP to generate tension at the AJC that restricts protrusion formation by ∆*inlC* mutant bacteria [39]. As described in Section 2, Dynamin 2 contains a carboxyl-terminal PRD domain with proline-rich sequences (Figure 3) [62]. Tijoriwalla et al. (2024) found that the PRD in Dynamin 2 binds directly to an amino-terminal region in Tuba called “SH31-4” that contains four tandem SH3 domains (Figure 5) [39]. Several lines of evidence indicate that interaction of this SH31-4 region in Tuba with the Dynamin 2 PRD recruits the GTPase to tight junctions (Figure 6A) where it contributes to cortical tension that limits protrusion formation of a ∆*inlC* mutant strain of *L. monocytogenes* (Figure 6B). First, confocal microscopy imaging demonstrates that Tuba and Dynamin 2 co-localize at tight junctions. Secondly, RNA interference (RNAi)-mediated depletion of Tuba or deletion of the PRD in Dynamin 2 abolishes localization of the GTPase to these junctions. Thirdly, knockdown of Dynamin 2 by RNAi or inhibition of its GTPase activity using the compound dynasore [63] decreases cortical tension at the AJC and restores normal protrusion formation and cell-to-cell spread of the ∆*inlC* mutant *L. monocytogenes* strain.

An interesting and potentially important finding from the Tijoriwalla et al. (2024) study was that infection with wild-type *L. monocytogenes* or ectopic expression of InlC in Caco-2 cells resulted in the displacement of both Dynamin 2 and Tuba from tight junctions (Figure 6C) [39]. These results suggest that in uninfected cells, human ligands of the Tuba SH36 domain such as N-WASP might stabilize Tuba and/or Dynamin 2 at junctions. In this scenario, when *L. monocytogenes* expressing InlC disrupts interaction of N-WASP with Tuba, the scaffolding protein and Dynamin 2 both disengage from tight junctions. The end result is decreased intercellular tension and enhanced cell-to-cell spread of bacteria.

### 3.2. Outstanding Questions

The findings in this study reveal an important physiological function for Dynamin 2 in regulating tension at tight junctions and indicate that *L. monocytogenes* has evolved the ability to antagonize this GTPase to augment bacterial spread. How does Dynamin 2 contribute to cortical tension? One possibility is that the GTPase does so through its well-established functions in endocytosis and recycling of endocytosed proteins back to the plasma membrane [34]. Several tight junction components, including the transmembrane protein occludin, undergo constitutive endocytosis and recycling to maintain barrier function [64,65]. Occludin modulates cortical tension by interacting with the scaffolding protein ZO-1, which associates with actin filaments and myosin II proteins (Figure 4A) [57]. Since endocytosis of occludin depends on Dynamin 2 [65], it is possible that this GTPase contributes to tension by mediating the internalization and subsequent recycling of occludin back to tight junctions (Figure 4B).

In future studies, it will be important to determine the extent to which the endocytic function of Dynamin 2 affects junctional tension and spread of *L. monocytogenes*. Considering the known roles of Dynamin 2 in clathrin- and caveolin-mediated endocytosis [37], the effects of inhibition of these endocytic pathways on tension and *L. monocytogenes* spread could be tested using chemical inhibitors and/or RNAi to target specific pathway components [66,67].

## 4. Dynamin 2 Regulation of DMV Escape by *Shigella flexneri*

### 4.1. Role of the Host Kinase PIK3C3 and Dynamin 2 in Cell-to-Cell Spread of S. flexneri

**Figure 7:**
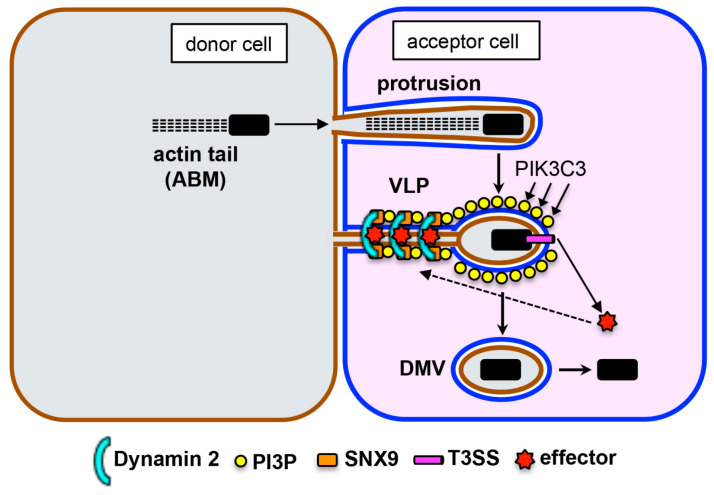
Role of Dynamin 2 in resolution of *S. flexneri* VLPs to DMVs. After conversion of protrusions to VLPs, the host kinase PIK3C3 synthesizes the lipid phosphatidylinositol 3-phosphate (PI3P), which decorates the donor cell membrane of VLPs. Dynamin 2 is recruited to the neck of VLPs and mediates their conversion to DMVs. While PI3P is required for mobilization of Dynamin 2 to VLPs, the broad distribution of this phosphoinositide on VLPs does not explain why Dynamin 2 is concentrated at the neck of these structures. Accumulation of Dynamin 2 at the VLP neck may involve the host protein SNX9 and/or one or more T3SS effector proteins translocated into the acceptor cell.

*S. flexneri* is a Gram-negative bacterial pathogen that causes bacterial dysentery (bloody diarrhea), resulting in 200,000 annual deaths worldwide [7,68]. This bacterium infects colonic epithelial cells, gains access to the host cytosol, and uses ABM to initiate intercellular spread. Experiments using the human colonic cell line HT-29 have revealed that protrusions produced by *S. flexneri* convert to “vacuole-like protrusions” (VLPs) characterized by a thin membrane stalk that lacks actin and is tethered to the plasma membrane (Figure 2B) [69]. Until recent work by Rolland et al. (2025) [40], how VLPs resolve into DMVs was not well understood. These authors found that resolution requires accumulation of the lipid phosphatidylinositol 3-phosphate (PI3P) on the VLP membrane of host cells that accept protrusions [40] (Figure 7). Moreover, this accumulation of PI3P is accompanied by recruitment of Dynamin 2 to the neck of VLPs and the successful conversion of these structures into DMVs.

The authors uncovered a role for PI3P in protrusion resolution by performing a chemical screen for host kinases needed for cell-to-cell spread of *S. flexneri*. These studies led to the discovery that the compounds VPS34-IN1 and SAR405, both inhibitors of the human class III phosphoinositide 3- kinase PIK3C3 [70], impair intercellular spread of bacteria in HT-29 and Caco-2 cells [40]. Experiments involving RNAi-mediated depletion of PIK3C3 corroborated these inhibitor-based findings. The authors then performed live imaging studies using spinning disc confocal microscopy. Using a fluorescently tagged probe that specifically recognizes PI3P, it was found that this lipid accumulates on VLPs in HT-29 cells accepting these structures, remains associated with bacteria during the transition of VLPs to DMVs, and then disappears shortly after DMV formation. As expected, treatment of host cells with VPS-IN1 abrogated recruitment of the PI3P probe to VLPs. Importantly, this treatment also strongly impaired progression of VLPs to DMVs, resulting in a ~90% decrease in release of bacteria into the cytosol of neighboring cells.

Subsequent experiments showed that, in the absence of treatment with VPS-IN1, fluorescently tagged Dynamin 2 was recruited to the neck of VLPs immediately prior to their conversion to DMVs (Figure 7). By contrast, inhibition of PI3P synthesis using VPS-IN1 impaired Dynamin 2 recruitment, demonstrating that accumulation of PI3P on VLPs is critical for this event. Depletion of Dynamin 2 by RNAi caused an approximately 40% inhibition in cell-to-cell spread of *S. flexneri*, which confirmed previous findings that this GTPase is needed for efficient spread [30,71]. However, one surprising aspect of the Rolland et al. study is that it did not address if Dynamin 2 is needed for progression of VLPs to DMVs. In principle, such experiments could be performed by inhibiting Dynamin 2 GTPase activity using compounds such as dynasore, Dyngo 4a, or Dyngo 6a [63,72] or by RNAi-induced knockdown of Dynamin 2. Notwithstanding this comment, the collective results by Rolland et al. (2025) [40] provide convincing evidence that Dynamin 2 contributes to the conversion of VLPs to DMVs.

The authors used an infant rabbit model to assess the role of PIK3C3 in infection in vivo. This model recapitulates many of the features of disease (shigellosis) in humans, including bloody diarrhea, epithelial fenestration, and intercellular spread in the colon [73]. Compared to control rabbits, animals treated with the PIK3C3 inhibitor SAR405 exhibited decreased sizes of foci of infection in the colonic epithelium. Importantly, recruitment of F-actin by bacteria was unaffected by drug treatment, indicating a lack of effect on ABM. The results support the findings with cultured human cells and provide evidence that PIK3C3 acts after ABM to control cell-to-cell spread of *S. flexneri* in vivo.

### 4.2. Outstanding Questions

How is Dynamin 2 mobilized to VLPs to stimulate their resolution? Rolland et al. (2025) [40] did not examine the mechanism by which PI3P recruits Dynamin 2 to VLPs. While the PH domain of Dynamin 2 interacts with PI3P, its binding affinity for this phosphoinositide is about 10-fold lower than for PI (4,5)P_2_ [74]. This low affinity, combined with the observation that PI3P localizes to VLPs before Dynamin 2, suggests that PI3P alone is insufficient for mobilization of Dynamin 2. The authors suggest that the sorting nexin protein SNX9 might work together with PI3P to mediate Dynamin 2 recruitment (Figure 7). SNX9 has an amino terminal SH3 domain that binds Dynamin 1 and 2 proteins, a PX domain that interacts with PI3P, and a concave-shaped BAR domain that recognizes positive membrane curvature [75,76,77]. The authors speculate that recruitment of Dynamin 2 to the neck of VLPs might be mediated by coincident binding of the PX domain of SNX9 to PI3P, its BAR domain to the VLP neck, and its SH3 domain to Dynamin 2 (Figure 7). Another interesting idea is that PI3P and SNX9 might enhance the membrane fission activity of Dynamin 2 to resolve VLPs into DMVs. PI3P is known to synergize with SNX9 to stimulate Dynamin GTPase activity, which is essential for membrane fission [76].

Do T3SS effectors of S. flexneri contribute to recruitment of Dynamin 2? *S. flexneri* uses a molecular syringe called a type III secretion system (T3SS) to produce pores in the host cell plasma membrane that allow the delivery of ~25 bacterial effector proteins into the cytoplasm of human cells [7]. Many of these effectors control various steps of the intracellular life cycle of *S. flexneri*, including internalization, vacuolar escape, protrusion formation and resolution, and DMV escape. Could an effector protein be translocated into a protrusion-accepting host cell and work together with PI3P to mobilize Dynamin 2 for protrusion resolution? Recent results show that the *S. flexneri* T3SS sequentially makes pores in the two membranes of the DMV, and that the ability to generate these pores is needed for bacteria to escape this double membranous vacuole [78]. These findings raise the possibility that the T3SS might also produce pores in the two VLP membranes, resulting in delivery of effectors into neighboring cells to recruit Dynamin 2 to resolve VLPs (Figure 7).

## 5. Dynamin 2’s Role in Spread of *Burkholderia thailandensis*

### 5.1. Introduction

The *Burkholderia* species *pseudomallei* is a Gram-negative bacterium that causes melioidosis, characterized by pneumonia, sepsis, and abscess formation with a mortality rate of 10–50% [8,79]. The species *Burkholderia thailandensis* is less virulent than *B. pseudomallei* and is used as a model for this latter species in experiments infecting cultured cell lines or animals. Both *B. pseudomallei* and *B. thailandensis* induce their internalization into human cells, escape from membrane vacuoles to replicate in the host cytosol, and undergo ABM to spread to neighboring cells [8,17,18,80,81] (Figure 1). These two bacterial species each have a type VI secretion system (T6SS) referred to as “T6SS-5” that is needed for the formation of multinucleated giant cells (MNGCs), which result from fusion of the plasma membrane of infected cells with those of neighboring cells [81,82,83,84]. MNGCs have been detected in patients with melioidosis and in mice infected with *B. pseudomallei* [81], suggesting that they may contribute to disease.

Cell–cell fusion mediated by T3SS-5 was thought to be the sole mechanism by which intercellular spread of *Burkholderia* spp. occurs. However, recent work has revealed that, prior to MNGC formation, *B. thailandensis* produces plasma membrane protrusions that contribute to spread [19,20]. Plum et al. (2024) showed that Dynamin 2 promotes resolution of *B. thailandensis* protrusions by detaching these structures from host cells (Figure 2D) [20]. Subsequently, the bacterium’s T6SS-5 promotes lysis of severed protrusions, which seem similar to DMVs. This lysis liberates bacteria into the cytoplasm of a neighboring cell and allows bacteria to rapidly undergo ABM for a new cycle of infection.

### 5.2. Role of Dynamin 2 in Cell-to-Cell Spread of B. thailandensis

A major goal of the Plum et al. (2024) [20] study was to investigate the dynamics of assembly of the *B. thailandensis* T6SS-5 system during cell-to-cell spread in cultured human cells. By infecting the human lung epithelial cell line A549 with *B. thailandensis*, the authors found that intracellular bacteria were frequently observed outside of MNGCs [20]. In addition, a *B. thailendensis* mutant strain deleted in a gene encoding a critical T6SS-5 component failed to induce MNGC formation but still exhibited cell-to-cell spread. Since a previous study showed that *B. thailandensis* makes protrusions while spreading [19], Plum et al. (2024) [20] set out to examine assembly of T6SS-5 in protrusions.

**Figure 8:**
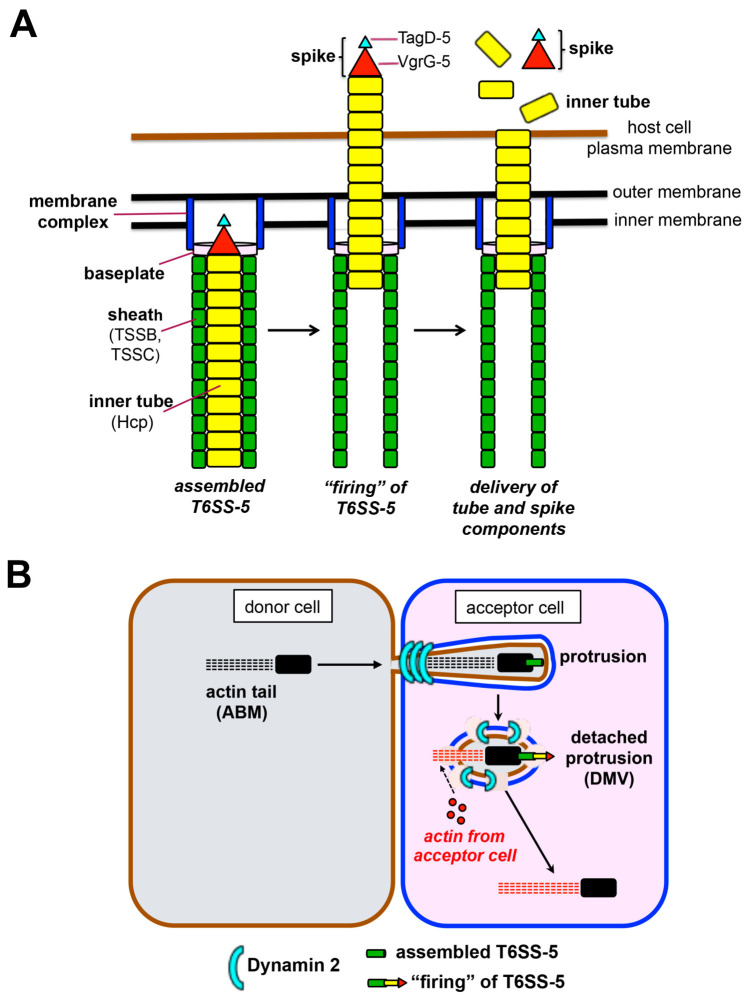
Manipulation of Dynamin 2 by *B. thailandensis* to promote detachment and lysis of protrusions during spread. (**A**) T6SS-5 of *B. thailandensis*. Upon contact with host cells, the contracted inner tube and spike complex are propelled to perforate the plasma membrane of human cells. In addition to disrupting this membrane, the spike complex and tube components may exert effector functions to modulate processes in the host cytosol. (**B**) Role of Dynamin 2 in detachment and lysis of protrusions. Upon assembly of the T6SS-5 in protrusions, Dynamin 2 is mobilized to the base of these structures and mediates their fission from the plasma membrane. The T36SS-5 is assembled again in the detached protrusion, which is physically resembles a DMV. Following assembly of T6SS-5, Dynamin 2 is recruited to the detached protrusion, which then lyses. Actin monomers from the cytosol of the acceptor host cell are polymerized to make an actin tail, which may contribute to further rupturing of the DMV and subsequent bacterial escape.

T6SSs are widespread in Gram-negative bacteria and act as a multi-protein contractile apparatus that delivers effector proteins into target cells, which may be bacterial or eukaryotic [85,86]. The T6SS-5 of *B. pseudomallei* and *B. thailandensis* targets human cells of various types, including phagocytes and epithelial cells of the upper and lower respiratory tract [8,18,81]. Like other T6SSs, the T6SS-5 comprises a contractile sheath surrounding an inner tube in the cytoplasm of the bacterium [85] (Figure 8A). The sheath and inner tube are capped with a baseplate and spike complex. Contraction of the sheath “fires” the T3SS6-5, propelling the spike complex and part of the inner tube into the target host cell. This action punctures the host cell plasma membrane and delivers inner tube components and the spike complex into the cytosol.

To investigate assembly of T6SS-5, the authors used a construct in which the sheath component TssB was fused to the fluorescent protein mScarlet-I [20]. Spinning disc microscopy imaging of infected human HeLa cells showed that localization of fluorescent TssB was dynamic, frequently clustering into foci at cell poles, and then exhibiting diffuse cytoplasmic localization. These localization patterns are thought to indicate assembled and disassembled T6SSs. About 80% of assembled T6SS-5 events in HeLa cells were detected in protrusions, whereas the remainder were present in cytosolic bacteria or bacteria contacting areas of the plasma membrane outside of protrusions. These results indicated that T6SS-5 assembly occurs preferentially in protrusions.

Experiments involving labeling of the human lung epithelial cell line A549 with the plasma membrane stain CellMask revealed that protrusions made by wild-type *B. thailandensis* were initially covered by the plasma membrane of the host cell. However, 30% of these protrusions later underwent membrane lysis, usually after detachment from the protrusion-donating cell (Figure 8B). In ~80% of cases, lysis was immediately preceded by assembly of the T6SS-5 in protrusions. By contrast, bacterial mutant strains inactivated in T6SS-5 genes exhibited lysis of only 8% of protrusions. These results indicate that most lysis events are triggered by assembly of the T6SS-5. Importantly, only 18% of cells with lysed protrusions fused to form MNGCs. Collectively, these findings show that the main mechanism of cell-to-cell spread occurs through protrusion lysis, rather than MNGC formation.

Given that Dynamin 2 was previously found to be required for cell-to-cell spread of *S. flexneri* [30,71], Plum et al. performed a series of experiments to investigate the role of this GTPase in spread of *B. thailandensis*. Treatment of A549 cells with dynasore, a chemical inhibitor of Dynamin GTPases [63], reduced protrusion lysis more than two-fold, without affecting MNGC formation [20]. Similar results were observed in a mouse fibroblast cell line that had knockout mutations in the genes for all three Dynamin proteins. The authors then examined localization of Dynamin 2 throughout the course of protrusion formation and lysis. After assembly of the T6SS-5 in protrusions, Dynamin 2 was recruited to the base of these structures, followed by detachment of the protrusion from the host cell about 2 min later (Figure 8B). The physical appearance of these detached protrusions resemble DMVs observed with *S. flexneri* or *L. monocytogenes*. Shortly after detachment, the T6SS-5 assembled again in the internalized protrusion, followed by immediate recruitment of Dynamin 2 and membrane lysis. Interestingly, lysis was accompanied by polymerization of actin comet tails in bacteria in detached protrusions, with the host cell accepting the protrusion providing actin monomers for this polymerization (Figure 8B). Collectively, these results provide evidence that the T6SS-5 induces recruitment of Dynamin 2 to mediate protrusion detachment. How lysis of detached protrusions is accomplished was not directly addressed in this study. However, it seems plausible that the membrane-puncturing activity of T6SS-5 [85], combined with the force provided by ABM, contribute to lysis. While Dynamin 2 is needed for lysis of detached protrusions, it is unclear if this role is direct or instead indirectly due the GTPase’s function in the prior step of protrusion detachment.

Interestingly, evidence was presented that lysed protrusions of *B. thailandensis* fail to trigger a host autophagy response. The host protein galectin-3 binds to β-galactosides on damaged vacuoles of several intracellular bacterial pathogens including *L. monocytogenes*, *Legionella pneumophila*, *S. flexneri*, and *Yersinia pseudotuberculosis* [87,88,89]. Since galectin-3 can promote autophagy [90], the authors examined its recruitment to detached protrusions containing *B. thailandensis*. Whereas lysis of wild-type *B. thailandensis* protrusions was accompanied by galectin-3 recruitment in only 3% of cases, null mutations in components of T6SS-5 increased the recruitment frequency by 7–20 fold [20]. Similarly, the autophagy protein LC3 was mobilized to only 0.40% of intracellular wild-type *B. thailandensis*, and this frequency was augmented 10-fold for a mutant strain inactivated in T6SS-5. These results suggest that protrusion lysis mediated by T6SS-5 might allow evasion of autophagy. However, this idea was not explored further through experiments demonstrating that autophagy components restrict intracellular replication of *B. thailandensis* strains mutated in T6SS-5 genes.

### 5.3. Outstanding Questions

How is Dynamin 2 recruited to protrusions to mediate their detachment? Given that T6SS-5 assembles in protrusions immediately prior to recruitment of Dynamin 2, this secretion system would be expected to be required for mobilization of Dynamin 2 to protrusions. Testing this idea could be accomplished by determining if *B. thailandensis* mutants inactivated in genes encoding T6SS-5 components fail to recruit Dynamin 2. It would be of particular interest to test the roles of the T6SS-5 spike components VgrG-5 and TagD-5 and the tube component Hcp-5 in Dynamin 2 recruitment (Figure 8A) [81]. In some bacterial species, these spike and tube components serve as effectors to module host function [85]. Importantly, Vgr5 and TagD-5 are needed for formation of MNGCs, suggesting that they may have effector functions for *B. thailandensis* [19,83].

Another important question is what function does the second recruitment of Dynamin 2 to detached protrusions serve? An interesting idea is that this event might contribute to evasion of autophagy. Dynamin 2 is known to interact with LC3 and mediate the fission of autophagosome precursors from an endomembrane compartment termed the recycling endosome (RE) [36,91]. Conditions that sequester Dynamin 2 at the plasma membrane inhibit the release of autophagosome precursors from the RE and their maturation to autophagosomes [36]. An intriguing possibility is that sequestration of Dynamin 2 in detached protrusions might contribute to the avoidance of autophagy by *B. thailandensis*. It might be possible to test this idea by determining if inhibition of Dynamin 2 with dynasore abrogates recruitment of LC3 to lysed protrusions. Although dynasore treatment reduces protrusion lysis, approximately 10% of protrusions still lyse in dynasore-treated cells, suggesting that such an experiment might be feasible.

## 6. Therapeutic Potential of Targeting Dynamin 2 or Bacterial Factors That Control This GTPase

The ability of *L. monocytogenes*, *S. flexneri*, and *B. thailandensis* to manipulate Dynamin 2 activity to promote intercellular spread raises the possibility that targeting Dynamin 2 with drugs might be an effective strategy to treat infections. In this regard, it is worth noting that the Dynamin family protein inhibitor Dyngo-4a has been used at doses of up to 30 mg/kg in mice without toxicity [92]. Similarly, the PIC3C3 inhibitor SAR405, which perturbs Dynamin 2 localization, was used in infant rabbits to demonstrate a role for PIK3C3 in *S. flexneri* infection (Rolland et al., 2025) [40] and in mice to study the relationship between autophagy and memory loss [93]. Taken together, these in vivo studies with Dyngo-4a and SAR405 suggest that inhibitors of Dynamin 2 or its localization may have the potential to treat bacterial infections. However, considerable work would need to be done to confirm lack of toxicity, develop effective delivery approaches, and test efficacy of treatment in humans.

In principle, inhibiting bacterial virulence factors that affect Dynamin 2 may provide a more specific and perhaps less toxic therapeutic strategy compared to direct targeting of Dynamin 2 itself. However, the design and delivery of such therapies would be challenging. In the case of *L. monocytogenes*, it might be possible to develop cell-permeable peptides or nanobodies [94] that interact specifically with a region in the virulence protein InlC that binds its human cytoplasmic receptor Tuba. This InlC region, referred to as the “leucine-rich-repeat” (LRR) domain interacts with the C-terminal SH36 domain in Tuba [58], resulting in the displacement of N-WASP from Tuba and the disassociation of Tuba and Dynamin 2 from tight junctions [39] (Figure 6). Drugs that block interaction of InlC with the SH3 domain in Tuba might therefore stabilize Dynamin 2 at tight junctions, which would be expected to attenuate cell-to-cell spread of *L. monocytogenes*. Similarly, drugs that affect the structure and function of T6SS-5 of *Burkholderia* spp. might be useful in treating infections with *B. thailandensis* or *B. pseudomallei*. One potential target for such drugs is the ATPase ClpV, which mediates the recycling of T6SS-5 components [85]. In the case of *S. flexneri*, bacterial proteins that manipulate host Dynamin 2 or PIK3C3 have yet to be identified. Therefore, further research is required to identify potential virulence proteins to target to impair Dynamin 2 function.

## 7. Conclusions

Recent investigations indicate that the human GTPase Dynamin 2 plays distinct roles in cell-to-cell spread of the bacterial pathogens *Listeria monocytogenes*, *Shigella flexneri*, and *Burkholderia thailandensis*. These roles include generating cortical tension to limit protrusion formation of *L. monocytogenes*, promoting the resolution of *S. flexneri* VLPs to double membranous vacuoles, and contributing to lysis of protrusions of *B. thailandensis*. Some unresolved questions to answer in future work include the following: (1) Does Dynamin 2’s endocytic activity contribute to cortical tension that limits spread of *L. monocytogenes*, (2) how does the GTPase promote lysis of *B. thailandensis* protrusions, (3) how is Dynamin 2 recruited to VLPs of *S. flexneri*, and (4) does Dynamin 2 control spread of SFG *Rickettsia* through effects on cortical tension, protrusion resolution, and/or DMV escape?

## Data Availability

No new data were created or analyzed in this study.
